# Hypopigmented Mycosis Fungoides in an 11-Year-Old Palestinian Boy

**DOI:** 10.1155/2023/4310796

**Published:** 2023-02-16

**Authors:** Duha Rabi, Balqis Shawer, Ahmad Rabee, Mohammad Qudaimat, Mohammad Milhem, Izzeddin Bakri

**Affiliations:** ^1^Al Quds University, Faculty of Medicine, Hebron, State of Palestine; ^2^Palestine Polytechnic University, Faculty of Medicine, Hebron, State of Palestine; ^3^Makassed Islamic Charitable Hospital, Department of Pathology, Jerusalem, Israel

## Abstract

Cutaneous T-cell lymphoma (CTCL) is a lymphoproliferative disorder of the skin. The most common subtype of CTCL in pediatrics is mycosis fungoides (MF). There are multiple variants of MF. The hypopigmented variant represents more than 50% of MF cases in pediatrics. Misdiagnosis of MF can occur because it may resemble other benign skin pathologies. This is a case of an 11-year-old Palestinian boy presented with generalized nonpruritic hypopigmented maculopapular patches with progressive course for 9-months. Biopsy samples from a hypopigmented patch revealed appearances diagnostic of MF. Immunohistochemical staining was positive for CD3 and CD7 (partial) and a mixture of CD4 and CD8 positive cells. The patient's case was managed with narrowband ultraviolet B (NBUVB) phototherapy. After a few sessions, the hypopigmented lesions improved significantly.

## 1. Introduction

Cutaneous T-cell lymphoma is the most common type of primary cutaneous lymphomas. It is a term that is used to identify a group of lymphoproliferative disorders that involve the skin [1]. The most common subtype of CTCL is mycosis fungoides [2, 3]. A study to describe the incidence of CTCL in the United States found that the incidence of MF is 6.4 per 1,000,000 every year in adults, but the occurrence in children is rare and has not been well established [4]. Although rare, it has been reported that MF is the most common form of CTCL in children [5].

A lot of clinical forms of mycosis fungoides were reported in the literature. The hypopigmented variant comprises more than half of MF cases in pediatrics [6]. The differential diagnosis of hypopigmented MF is vast; thus, delayed diagnosis of the condition is common. Misdiagnosis with other benign skin conditions may also occur [5].

Most literature focused on the diagnosis of MF in pediatrics, and lower attention towards the treatment and prognosis of the disease was given [7].

This case aims to document the occurrence of juvenile MF in Palestine and identify the method of treatment used and its effectiveness. We hope that this case will help raise awareness of the disease by illustrating the importance of early clinical suspicion of the condition.

## 2. Case Presentation

**Figure 1:**
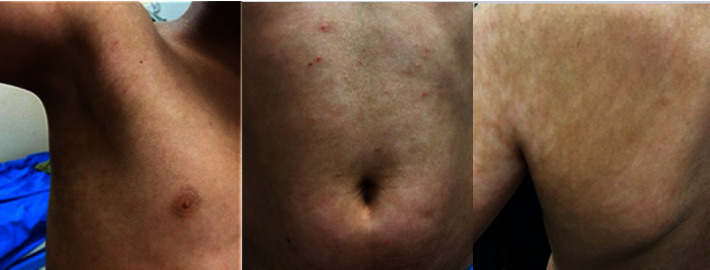
Hypopigmented patches involving the axilla, back, and abdomen.

An 11-year-old boy presented at the Dermatology Center at Palestine Medical Complex in Ramallah on account of 9 months to 1 year history of nonpruritic hypopigmented maculopapular patches that started on the axilla and then spread to the back with a progressive course until they involved most of his body surface area Figure 1. There was no history of myalgia, radiation, or chemical exposure, no swellings in his body, and no bleeding into his skin.

The past medical history of the patient is unremarkable, with no evidence of atopy, recent infections, other inflammatory dermatitis, or any relevant environmental exposure. The family history of the patient was free except for leukemia in a paternal uncle and two paternal cousins.

**Figure 2:**
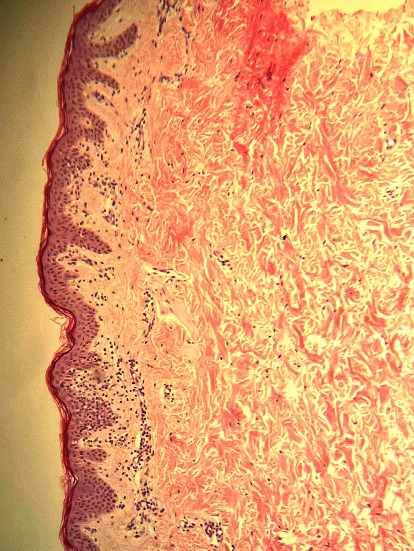
H & E 10x histopathology showing epidermotropism with occasional characteristic intraepidermal collections of atypical cells.

He was first prescribed emollients, with no benefit. However, due to the persistence and progression of the hypopigmented lesions, the patient underwent multiple punch biopsy samples from a hypopigmented patch from his trunk and ileal region, which were sent for histopathology. Skin biopsies showed superficial dermal and perivascular lymphocytic infiltrate. The epidermis showed scattered dyskeratotic keratinocytes and a few intraepidermal hyperchromatic atypical lymphocytes (Figure 2).

**Figure 3:**
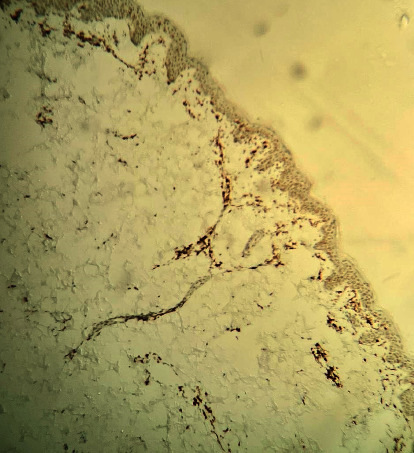
Immunohistochemical staining showing CD 4+ T cells.

**Figure 4:**
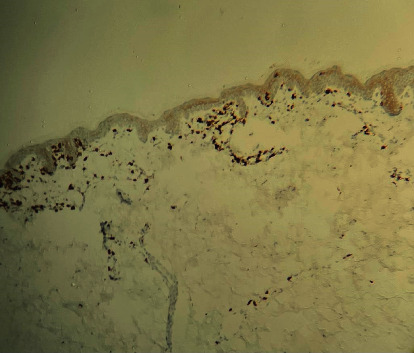
Immunohistochemical staining showing CD 8+ T cells.

Immunohistochemical staining was positive for CD3 and CD7 (partial) and a mixture of CD4 and CD8 positive cells with CD8 positive T lymphocytes predominance in neoplastic epidermotropism (Figures 3 and 4).

The stage at which the patient was diagnosed is stage 1B (T2 N0 M0 B0) according to the modified tumor-node-metastasis-blood (TNMB) classification. He initiated treatment with narrowband ultraviolet B (NBUVB) phototherapy sessions. The treatment was started in September, with 3 sessions weekly which continued for three months. In January, the sessions were decreased to twice a week. The treatment was stopped in March after approximately 6 months of therapy when the patient showed a great response to NBUVB light. The phototherapy sessions were decreased to once weekly in the last two to three weeks of treatment.

The patient is now being followed up closely for any recurrence of skin lesions.

## 3. Discussion

Pediatric age group mycosis fungoides has been reported scantly in literature. Literature, especially data regarding clinical symptoms and treatment course. In our case, we describe a case of hypopigmented MF in a Palestinian child. MF in pediatrics tends to be under reported [8]. This is because at first, MF has a clinical and histopathological resemblance to other benign inflammatory disorders [9, 10]. These can include pityriasis alba, vitiligo, pityriasis versicolor, and postinflammatory hypopigmentation [2, 5]. Another reason for delayed diagnosis is physicians' reluctance to perform early skin biopsies in children [8]. In our presented case, diagnosis of the condition was made after approximately 9 months from the start of the dermatologic manifestations.

There are several clinical variants of pediatric MF. The hypopigmented subtype accounts for more than 50% of pediatric MF cases. Classic MF represents about 41% of all MF in pediatrics. Folliculotropic, poikilodermatous, and hyperpigmented MF are other clinical variants that can present in pediatrics but to a lesser extent [6].

A case series of 34 juvenile onset mycosis fungoides patients from the United States reported that the majority of the pediatric patients presented with clinical stages IA and IB, just as presented in our case. Presentation at later stages of the disease which manifests with lymph node and visceral organ involvement can occur but is exceedingly rare [11].

Although mycosis fungoides is a sporadic disease, there have been reports of familial occurrences of MF in some families [12]. It has been reported that there is strong linkage disequilibrium between MF and HLA II allotypes in some populations, which significantly indicates a genetic predisposition [13]. In 1 of the families where familial MF was reported, leukemia in a first degree relative was also reported. This might suggest that familial predisposition to hematologic malignancies can also include MF [13]. In our case, the patient's uncle, a second-degree relative, and 2 cousins, third-degree relatives, all had leukemia.

It was documented that hypopigmented MF in pediatrics showed CD8+ T cell predominance with a reduced CD4 : CD8 ratio [14, 15]. In our case, immunohistochemical staining showed CD8+ T cell predominance over CD4+ T cells.

There are various treatment options for pediatric MF. Phototherapy is the most common one. Psoralen with ultraviolet light (PUVA) and narrowband ultraviolet light (NBUV) are the modalities used. Phototherapy with PUVA light has been found to significantly improve the disease with the lowest percent of recurrence [7]. However, NBUV light is considered the first-line treatment due to its safety profile. Treatment with psoralen UV light can expose patients to increased amounts of UVA light and thus raise the risk of nonmelanoma skin malignancy in these patients [16]. Topical agents such as topical corticosteroids and retinoids can also be used in combination with phototherapy to treat pediatric MF [6].

**Figure 5:**
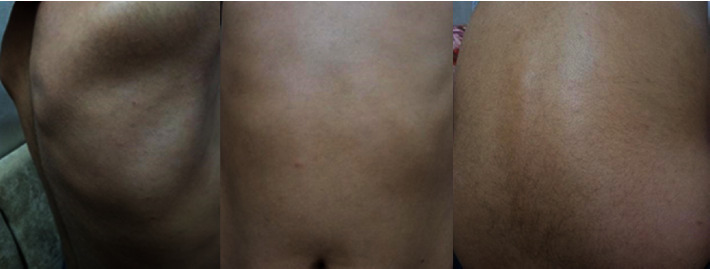
Significant improvement of the patient's symptoms after phototherapy.

In our case, NBUV light was used to treat the patient. It was reported that 71% of pediatric MF patients showed at least partial improvement with NBUV light therapy [16]. Response to phototherapy in MF patients counts on various factors; the specific variant of MF is an important one. In the presented case, hypopigmented MF shows an excellent initial response to NBUV light after approximately 6 months of phototherapy Figure 5.

## Data Availability

The data supporting this case report are from previously reported studies and datasets, which have been cited.
